# Effects of heat-treated *Lactobacillus gasseri* CP2305 on brain oscillatory activity and mental stress in healthy adults: a double-blind, randomized, crossover, placebo-controlled trial

**DOI:** 10.3389/fnut.2026.1796729

**Published:** 2026-05-28

**Authors:** Reiko Tanihiro, Ryoma Hosaka, Daisuke Sawada, Tatsuhiko Hirota

**Affiliations:** Core Technology Laboratories, Asahi Quality and Innovations, Ltd., Moriya, Japan

**Keywords:** brain oscillatory activity, gut-brain axis, lactobacillus, mental stress, postbiotic

## Abstract

**Background:**

Gut health and brain activity are closely linked, and the gut−brain axis plays a crucial role in regulating mental health. Interventions using beneficial microbes, such as probiotics and postbiotics, are gaining attention as promising approaches to support mental health. This study aimed to evaluate the effects of postbiotic heat-treated *Lactobacillus gasseri* CP2305 (CP2305) on mental stress and alpha oscillatory activity in the brain.

**Methods:**

This double-blind, randomized, crossover, placebo-controlled study included 28 healthy participants aged 20–44 years. The participants underwent two trial sessions and consumed a single dose of tablets containing heat-treated CP2305 (postbiotic; 1 × 10^10^ cells) or matching placebo tablets. In each session, electroencephalography (EEG) at eight positions was recorded from before to 60 min after ingestion to measure the alpha power. Heart rate variability was also recorded to calculate the root mean square of successive differences (RMSSD), an indicator of parasympathetic activity. The effects on mental state were evaluated using the visual analog scale (VAS) and Profile of Mood States, 2nd edition (POMS2). An *in vitro* study was conducted using the enterochromaffin cell line RIN14B to measure serotonin (5-HT) secretion levels following the heat-treated CP2305 administration.

**Results:**

The alpha power showed a significantly greater increase from baseline following consumption of heat-treated CP2305 compared with the placebo. The change in the RMSSD from baseline was significantly greater following consumption of heat-treated CP2305 than following the placebo. Both the mental stress VAS and POMS2 tension–anxiety scores showed significantly greater improvements following consumption of heat-treated CP2305 compared with the placebo. In the *in vitro* 5-HT assay, extracellular 5-HT levels were significantly higher under the heat-treated CP2305 condition than under the control condition.

**Conclusion:**

This is the first clinical study to examine the effects of postbiotics on alpha oscillatory activity in the brain. Heat-treated CP2305 consumption was associated with modulation of brain activity, enhancement of parasympathetic activity, and alleviation of stress, indicating its potential as a functional food ingredient for mental health support. These beneficial effects may be partly associated with alterations in gut-derived 5-HT signaling induced by heat-treated CP2305. However, further studies are required to elucidate the underlying mechanisms.

**Clinical trial registration:**

https://center6.umin.ac.jp/cgi-open-bin/ctr_e/ctr_view.cgi?recptno=R000064175, Identifier UMIN000056164.

## 1 Introduction

The brain serves as a control center for both mental and physical function. It orchestrates higher-level cognitive processes, such as thoughts, emotions, and memory, while managing vital bodily functions, such as heart rate, breathing, and movement (1, 2). Although the relationships between the brain, body, and mental activity are complex and many aspects remain unexplained, noninvasive brain monitoring technologies are beginning to shed light on these interactions. Brain waves recorded using electroencephalography (EEG) are composed of various waveforms at the different frequencies and are classified into alpha, beta, theta, delta, and gamma waves based on differences in their frequency and amplitude. The power of these frequency components varies depending on the mind–body state at a given time, including the levels of wakefulness, concentration, and relaxation, making them useful for understanding the mental and physical states. Alpha waves with a frequency of 8–13 Hz, an indicator of a relaxed state, represent a dominant EEG signature of eyes-closed rest and are typically predominant in the occipital region (3). Alpha power can be reduced by loaded visual stimuli and mental tasks, typically disappearing at sleep onset (4). Although the mechanism underlying alpha wave generation is not fully understood (5), the thalamus is thought to play a key role in generating alpha wave rhythms (6). Recent studies have suggested that activation of the thalamocortical loop may lead to increased alpha power in the occipital region (7). Alpha oscillatory activity has been reported to be activated by several visual, auditory, or olfactory treatments (8–11). Alpha power is activated not only when sensory organs receive stimuli from the external environment but also when ingesting certain food components. As an example of a food component, the oral intake of gamma-aminobutyric acid (GABA) has been reported to increase alpha power, and this effect is thought to be related to signaling between the gut and brain (12).

The brain communicates bidirectionally with the gut via the gut–brain axis (GBA), playing a role in various physiological and psychological processes required to maintain overall health (13). In the GBA, signals from the gut to the brain are transmitted via the nervous, endocrine, and immune systems. Within the neural transmission pathway, the vagus nerve, a crucial component of the parasympathetic nervous system, plays a key role (14). Over the past decade, several lactic acid bacteria have been reported to contribute to mental health by improving sleep quality or alleviating depression and anxiety (15, 16). These effects on mental health are thought to be mediated by the GBA. In a preclinical study, feeding *Lacticaseibacillus rhamnosus* JB-1 only alleviated depression- and anxiety-like behaviors in mice when the vagus nerve was intact (17). Several studies have reported that lactic acid bacteria activate the vagus nerve connecting the gut and brain. However, few studies have demonstrated the effects of lactic acid bacteria on brain oscillatory activity.

*Lactobacillus gasseri* CP2305 (CP2305) is a lactic acid bacterium, and postbiotic that is known to activate the parasympathetic nervous system, improve sleep quality, and alleviate stress (18–22). Postbiotics are defined as preparations of inanimate microorganisms and/or their components that confer health benefits on the host (23). Therefore, the bacterial components are thought to be involved in the expression of these functions. The psychological effects of bacterial components are known to involve mechanisms via the GBA, suggesting that their actions on the gut are transmitted to the brain through the GBA. To verify whether the signals induced by heat-treated CP2305 ingestion were transmitted to the brain, we conducted a double-blind, placebo-controlled, crossover trial to examine brain oscillatory activity.

## 2 Materials and methods

### 2.1 Test tablets

Heat-treated CP2305 powder was obtained as previously described (20). Tablets containing heat-treated CP2305 and placebo tablets were prepared using the same formulation, except for the presence or absence of heat-treated CP2305 (1 × 10^10^ cells per two tablets). This dose has been consistently used in previous clinical studies on heat-treated CP2305, in which various beneficial effects have been reported (18–22). To prepare the placebo tablets, heat-treated CP2305 powder was replaced with dextrin. Maltose, dextrin, starch, and vegetable oil were used to prepare both tablet types. The tablets containing heat-treated CP2305 and the placebo tablets were visually identical and indistinguishable in taste.

### 2.2 Study design and participants

This trial was conducted in Tokyo, Japan, from November to December 2024 as a randomized, double-blind, placebo-controlled, crossover study. This study was conducted according to the guidelines of the Declaration of Helsinki. The protocol was approved by the Public Health Research Center Foundation Ethics Review Committee for Research Involving Human Subjects (Tokyo, Japan; Registration No. 24 J0001). This study was registered in the University Hospital Medical Information Network (UMIN) Clinical Trials Registry (UMIN000056164). Participants were recruited between November and December 2024. Healthy Japanese individuals aged 20 to 44 years with a body mass index (BMI) of 18.5 or higher and less than 30 and no allergies to dairy products or fermented foods were included. Additionally, to be included in the study, the participants were required to have normal eyesight (including corrected eyesight) and hearing ability, not consume excessive amounts of alcohol or caffeinated beverages, not smoke excessively, and have no history of severe external head injury. The key exclusion criteria were as follows: (i) a history of cardiovascular disease, psychiatric disorder, gastrointestinal disorder, or serious disease; (ii) current treatment for injuries or illnesses; (iii) major health problems within the previous 2 months; (iv) adverse effects on social life associated with menstruation; (v) possible pregnancy, pregnancy, or lactation; and (vi) a principal investigator-determined ineligibility for participation. The participants were randomized in a 1:1 ratio to one of two sequences (placebo-CP2305 or CP2305-placebo) by generating a randomization table using the Python-based open-source PsychoPy package (24). The generation of the randomization table and allocation were performed by a person who was not involved in the data collection and analysis. Tablets containing heat-treated CP2305 and placebo tablets were packaged in code-labeled, visually identical pouches and used for the intervention. The participants, evaluators, and investigators were blinded to the allocation until the end of the trial.

**Figure 1:**
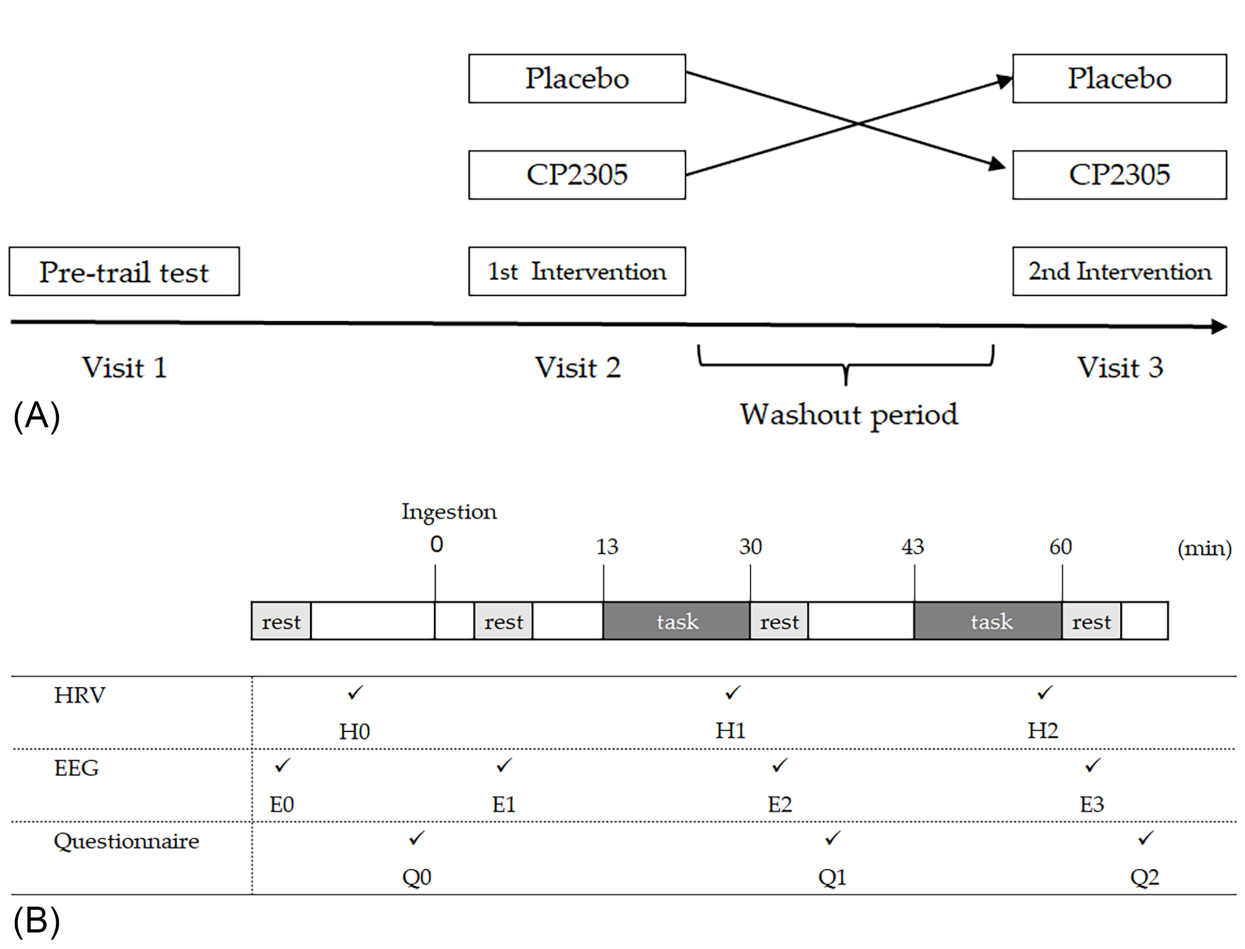
Study design **(A)** and experimental procedure at visits 2 and 3 **(B)**. H0–2: HRV measurements; E0–3: EEG measurements; Q0–2: questionnaire assessments. HRV: Heart rate variability. EEG: Electroencephalography.

This study included visit 1 (a pre-trial test), visit 2 (the first intervention), a washout period, and visit 3 (the second intervention; Figure 1A). At visit 1, participants answered the Zung Self-rating Depression Scale (SDS) (25) to obtain a baseline of characteristics related to depression and completed a series of experienced mental tasks and a visual analog scale questionnaire (VAS) to familiarize themselves with the trial in advance. At visits 2 and 3, the participants consumed a single dose of either heat-treated CP2305 or placebo. One group received placebo at visit 2 and heat-treated CP2305 at visit 3, whereas the other group received the interventions in the reverse order. At visits 2 and 3, EEG and heart rate variability (HRV) measurements and subjective assessments using questionnaires were performed. The two interventions were apart at least 3 days to avoid carryover effects. The washout duration in this study was set with reference to previous reports (12, 26) that evaluated the effects of a single-dose intervention on EEG alpha oscillatory activity. The primary outcome was defined as a change in the brain alpha power. Participants were instructed to avoid consuming lactic acid bacteria beverages or fermented foods containing lactic acid bacteria for 3 days prior to each visit and to refrain from consuming alcohol, caffeinated beverages, or health foods, smoking, and engaging in excessive exercise for 1 day prior to each visit. In addition, participants were required to abstain from food intake, except for water, for at least 3 h before EEG and HRV measurements.

### 2.3 Experimental procedure

At visits 2 and 3, the participants were first fitted with EEG caps and electrodes (Miyuki Giken Co., Ltd., Tokyo, Japan) and then directed to rest quietly for 5 min for acclimatization. After setting up the equipment and acclimatization, the experiment was performed as shown in Figure 1B. The procedures in this study were designed with reference to a previous report (12). First, the participants were instructed to close their eyes and rest for 4 min to measure their baseline EEG data (E0). Next, HRV measurements (H0) and subjective assessments (Q0) were performed to record the baseline conditions. After taking the test tablets (placebo or CP2305), the participants were instructed to close their eyes and rest for 4 min while their EEGs were recorded (E1). Mental tasks were performed during the 13–30-min and 43–60-min periods after ingestion. HRV measurements were obtained during the mental tasks (H1 and H2). After completion of the mental tasks, EEG measurements (E2 and E3) and subjective assessments (Q1 and Q2) were conducted sequentially. The protocol was programmed and controlled using PsychoPy (24) to guide the participants through the experimental procedure via a PC monitor. A photosensor attached to the monitor measured the screen brightness and generated a trigger at the start of each experimental stage to record the exact timing of each event.

The mental tasks included a mental arithmetic task (10 min) and an auditory oddball task (5 min) which were conducted in sequence with a 2-min rest between tasks. First, participants undertook the mental arithmetic tasks, which were programmed to automatically and continuously adjust difficulty based on their performance, with the aim of applying an appropriate cognitive load according to their abilities. Participants were instructed to sum two or more numbers displayed on a PC monitor in their mind and enter the results as quickly and accurately as possible using a numeric keypad. When the participant answered, or 18 s had passed without an answer, the screen automatically switched to the next problem. The calculation began with the addition of two double-digit numbers and one single-digit number. The program was designed such that the difficulty level increased each time two consecutive calculation problems were correctly answered. For example, if a participant answered six questions correctly in a row, the difficulty gradually increased from the initial setting (addition of two double-digit and one single-digit number) to the addition of three double-digit numbers, followed by the addition of three double-digit and one single-digit numbers, and further to the addition of four double-digit numbers. The program was designed such that the difficulty level gradually decreased each time two consecutive calculation problems were answered incorrectly.

In the second mental task, the participants performed an auditory oddball task using a two-tone paradigm. The target sound (2000 Hz, lasting for 0.1 s) and standard sound (1,000 Hz, lasting for 0.1 s) were randomly emitted at intervals of 0.8–1.2 s for 5 min, and their occurrence rates were 20 and 80%, respectively. The participants were instructed to respond only to the target sound and click the mouse button as quickly and accurately as possible. The experiments were conducted in a soundproof room.

### 2.4 EEG and HRV measurements

**Figure 2:**
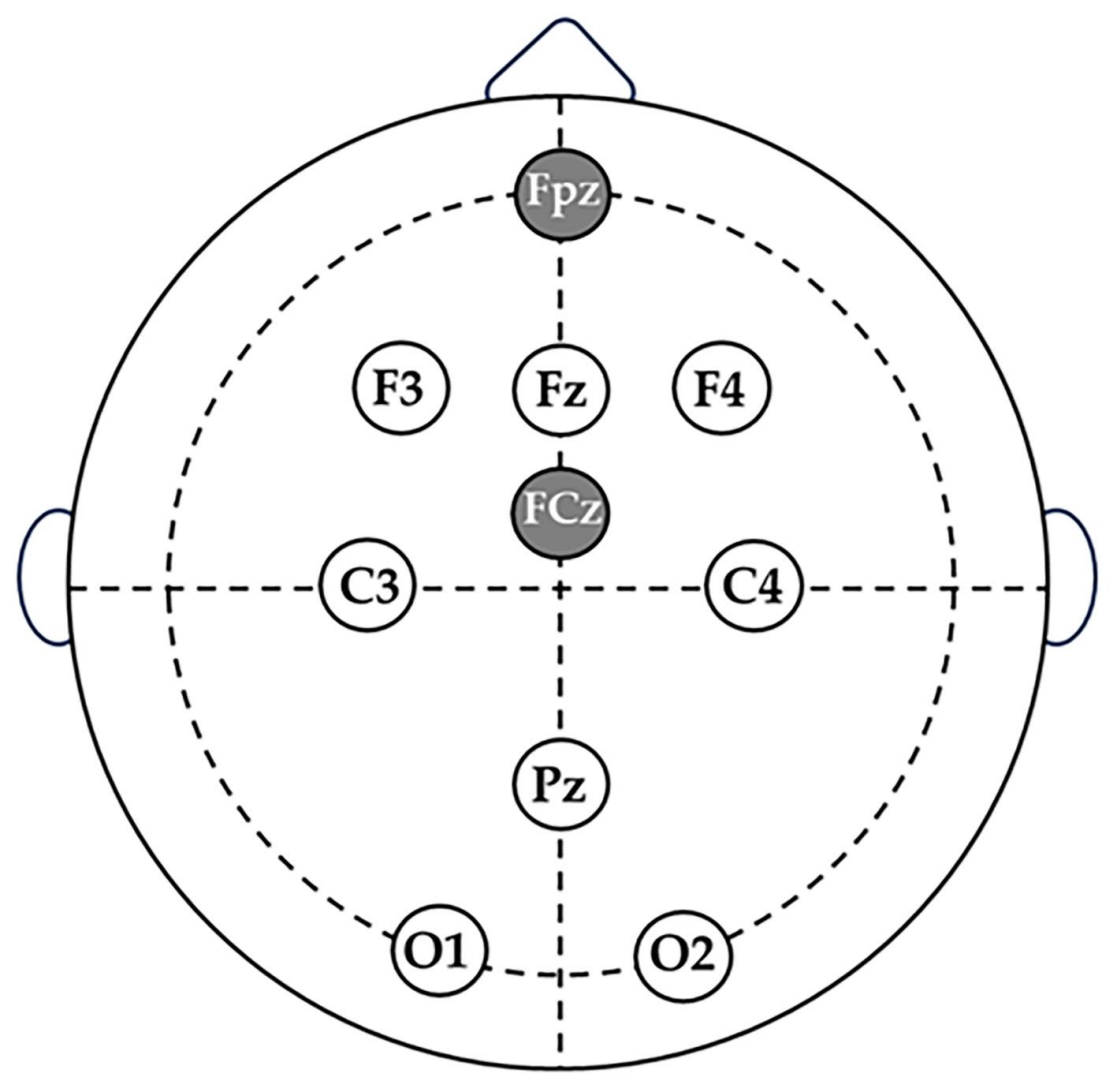
Placement of electrodes on the scalp. The ground electrode (Fpz) and reference electrode (FCz) are shown in gray.

EEG data were collected under eyes-closed conditions using an EEG cap with Ag/AgCl active scalp electrodes at the frontal (F3; left, Fz; midline, F4; right), central (C3; left, C4; right), parietal (Pz; midline), and occipital (O1; left, O2; right) positions based on the extended 10–20 system (Figure 2). Midline forehead (Fpz) and midline frontocentral (FCz) electrodes served as the ground and reference electrodes, respectively. To help remove ocular artifacts, such as eye movements and blinks, vertical and horizontal electrooculogram signals were obtained. Electrodes were attached to the fifth left intercostal space at the midclavicular line (V5 position) and the manubrium sternum to record cardiac activity using electrocardiography (ECG) with a bipolar CM5 lead. EEG and ECG data were recorded using Polymate® biological signal recording device (Miyuki Giken Co., Ltd., Tokyo, Japan) with a sampling rate of 1,000 Hz; the impedances of all electrodes were kept below 20 kΩ.

### 2.5 EEG and HRV analyses

MNE-Python, an open-source Python package, was used to analyze EEG data (27). The data were passed through a 1–50-Hz band-pass filter with a 50-Hz notch and downsampled to 250 Hz. An artifact exclusion procedure that automatically excluded data points exceeding 150 μV/ms was adopted. Independent component analysis was used to detect and remove artifacts derived from the ECG and electrooculography. The alpha power (8–13 Hz) and beta power (13–30 Hz) for each of the eight channels were calculated by a fast Fourier transform algorithm using Welch’s method with a 2-s Hanning window (50% overlap) (28). ECG signal preprocessing was performed using the Python-based open-source toolbox Neurokit 2 (29). The ECG data were passed through a 1–40-Hz band-pass filter. The obtained RR intervals were used to calculate the root mean square of successive differences (RMSSD), a time-domain measure of HRV. The RMSSD is calculated as the square root of the mean of the squared differences between successive RR intervals and primarily reflects short-term, vagally mediated parasympathetic activity. Higher RMSSD values indicate greater parasympathetic modulation and higher beat-to-beat HRV (30).

### 2.6 Questionnaires

The depressive characteristics of the participants at baseline were assessed using the SDS questionnaire (25). SDS scores can range from 20 to 80, with 20–39 indicating no signs of depression, while 40–47, 48–55, and 56–80 indicate mild, moderate, and severe depression, respectively (31). The degree of mental stress among participants was assessed using a VAS, which is 100 mm in length and anchored with “low mental stress” at 0 mm and “high mental stress” at 100 mm. The VAS score was calculated based on the distance from the 0 mm position, with a higher VAS score indicating higher stress. The Profile of Mood States 2nd Edition (POMS2)-adult shortened version (Kaneko Shobo Inc., Tokyo, Japan) was used to assess the mood changes of participants during the experimental procedure (32–34). The POMS2 consists of five negative mood scales (anger–hostility, confusion–bewilderment, depression–dejection, fatigue–inertia, and tension–anxiety) and two positive mood scales (vigor–activity and friendliness).

### 2.7 *In vitro* assay

RIN14B cells were obtained from American Type Culture Collection (ATCC, CRL-2059) and cultured in RPMI 1640 ATCC modified medium supplemented with 10% fetal bovine serum, 100 U mL^−1^ penicillin, and 100 μg mL^−1^ streptomycin at 37 °C with 5% CO_2_ in a humidified incubator.

*In vitro* assays were performed with reference to previously reported methods (35, 36). The heat-treated CP2305 powder was washed with Hank’s balanced salt solution (HBSS) containing calcium and magnesium (FUJIFILM Wako Pure Chemical Corporation, Osaka, Japan). After removing HBSS, washed the bacterial cells were resuspended in RPMI 1640 ATCC modified medium (3 mg mL^−1^) and homogenized. RIN14B cells were incubated in 24-well plates (1 × 10^5^ cells/well) for 24 h and washed with HBSS. Subsequently, HBSS was removed and replaced with RPMI 1640 ATCC modified medium containing heat-treated CP2305 (*n* = 5). Under control conditions, the replaced medium did not contain heat-treated CP2305 cells (*n* = 5). After further 24 h incubation, the medium was centrifuged for 15 min at 1,000 × *g*, and the supernatants were stored at −80 °C until 5-hydroxytryptamine (5-HT, serotonin) measurement. 5-HT concentration in the supernatant was measured using a serotonin ELISA kit (Enzo Life Science, Inc., Farmingdale, NY, United States).

### 2.8 Statistical analyses

The sample size for this clinical study was calculated based on a previous study (21). In that study, a partial eta squared value of 0.06 was reported for stress irritability, which corresponds to a medium effect size (*f* = 0.25). Using G*Power 3.1 (37), an *a priori* power analysis was conducted assuming an effect size of 0.25, an *α* error of 5%, and a statistic power of 80%, resulting in a required total sample size of 24. Considering the possibility of dropouts during the trial and electrical issues with the EEG measurements, the sample size was set to 28. Statistical analyses were performed using JMP version 13 software (SAS Institute Japan, Tokyo, Japan). A two-way analysis of variance (ANOVA) with repeated measures was used to compare the effects of the two interventions in the clinical study. Measurements at H0, E0, and Q0 were used to calculate post-intervention changes. Student’s *t*-test was used to compare the 5-HT concentrations in the *in vitro* study. Differences were considered statistically significant at *p* < 0.05.

## 3 Results

### 3.1 Demographics

**Figure 3:**
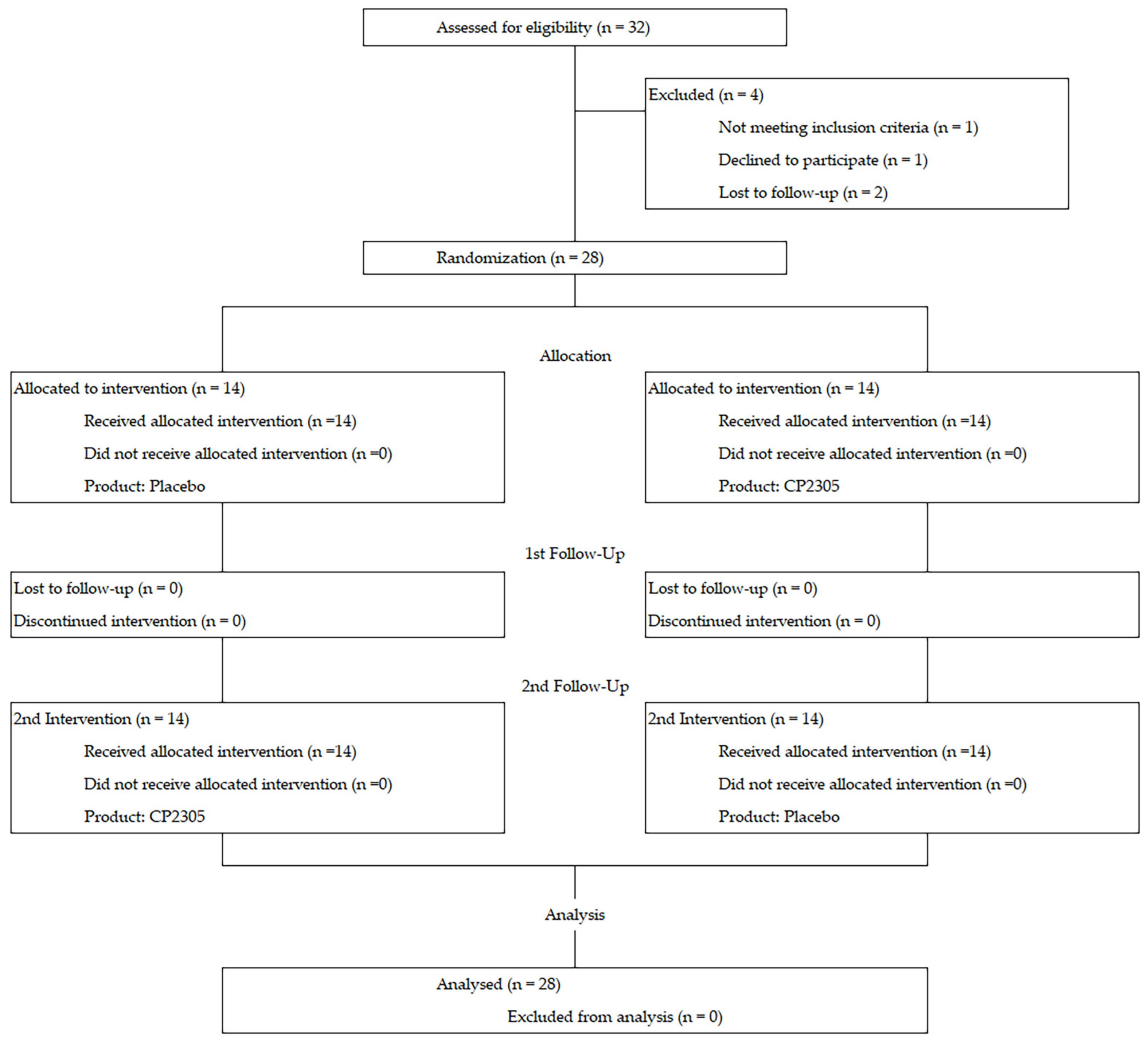
CONSORT flowchart.

A total of 32 individuals were recruited, of whom 28 were included in this study. Figure 3 shows the CONSORT flowchart of the study participants. All 28 participants completed the trial, and no adverse events or side effects were reported. The characteristics of the participants who completed the study are listed in Table 1. Trigger logs for the EEG and HRV data from four participants were not acquired because of faulty connections or battery malfunctions of the photosensor. These participants were excluded from the EEG and HRV analyses. One participant was excluded from the EEG analysis because of abnormal EEG patterns observed throughout the testing period. Specifically, the alpha power was lower during all eight eyes-closed periods (visits 2 and 3) than during the adjacent eyes-open periods. One-time point EEG data derived from another participant (E0 in visit 2, the placebo intervention) were excluded from the EEG analysis because of abnormal EEG patterns, where the average occipital alpha power was lower than the average frontal and central alpha power. EEG data derived from 23 participants (excluding E0 and changes at E1, E2, and E3 from E0 in the placebo condition from one participant) and HRV data derived from 24 participants were used in the final analysis. The results of the SDS assessment conducted at visit 1 confirmed that none of the participants had moderate or severe depressive characteristics at baseline.

**Table 1:** Characteristics of the participants (*n* = 28).

Variables	Units	Mean ± SD^1^
Age	years	34.6 ± 4.9
Weight	kg	60.7 ± 11.0
Height	cm	165.8 ± 8.4
BMI^2^	kg/m^2^	22.0 ± 2.8
Gender: Female / Male	*n* (%)	14 (50) / 14 (50)
Smoking: Yes / No	*n* (%)	2 (7) / 26 (93)
SDS^3^ score		34.6 ± 4.8

1 SD, standard deviation.

2 BMI, body mass index.

3 SDS, Zung Self-rating Depression Scale.

### 3.2 EEG and HRV

**Figure 4:**
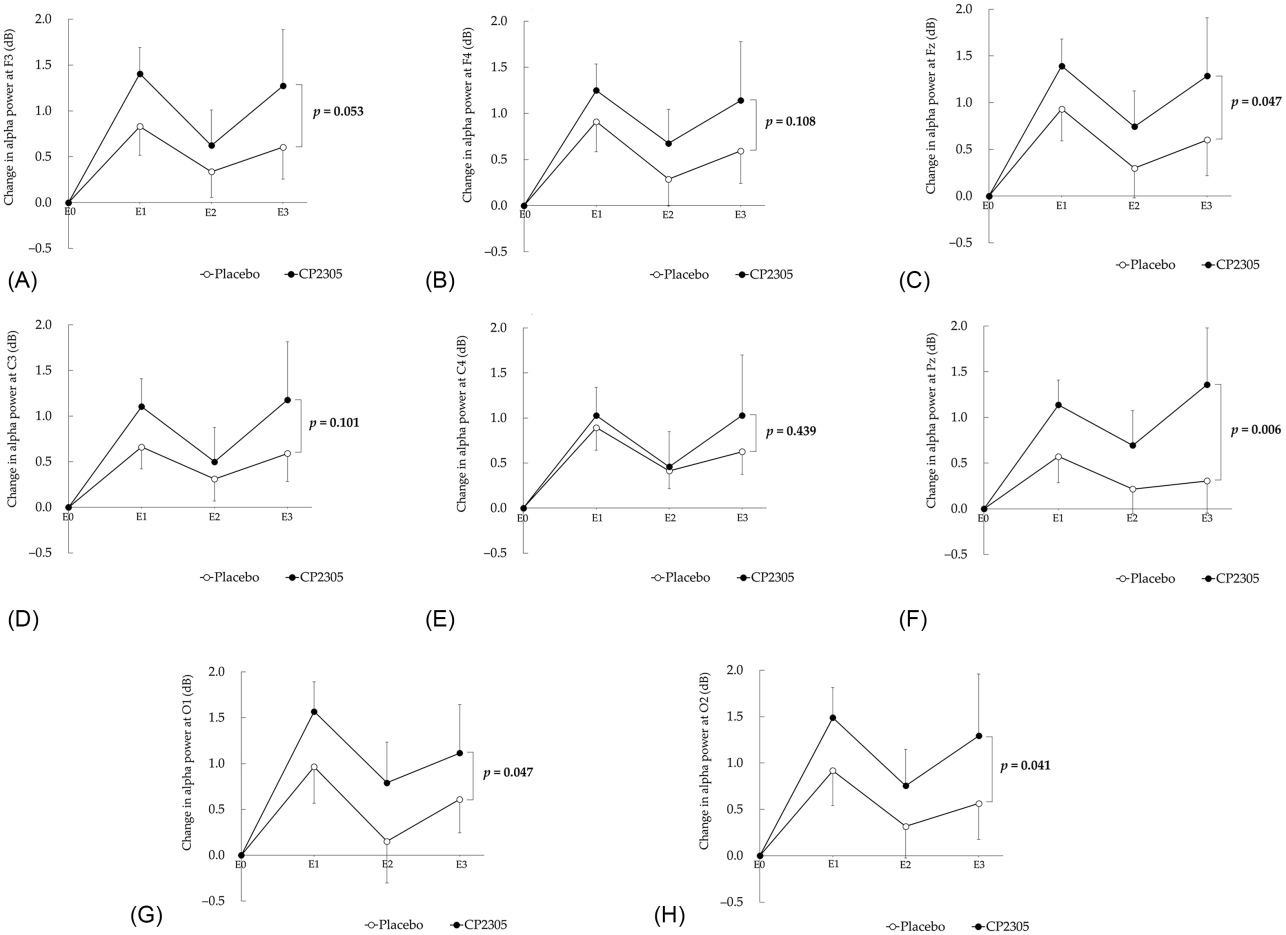
The effects of heat-treated CP2305 on the alpha oscillatory brain activity. Changes in alpha power from baseline (E0) at the eight electrodes were compared between the placebo (*n* = 22) and heat-treated CP2305 (*n* = 23) interventions. **(A)** F3 (left frontal) electrode, **(B)** F4 (right frontal) electrode, **(C)** Fz (middle frontal) electrode, **(D)** C3 (left central) electrode, **(E)** C4 (right central) electrode, **(F)** Pz (middle parietal) electrode, **(G)** O1 (left occipital) electrode, and **(H)** O2 (right occipital) electrode. Error bars represent standard errors. The *p*-values were calculated using a two-way ANOVA with repeated measures for the differences between the groups.

**Figure 5:**
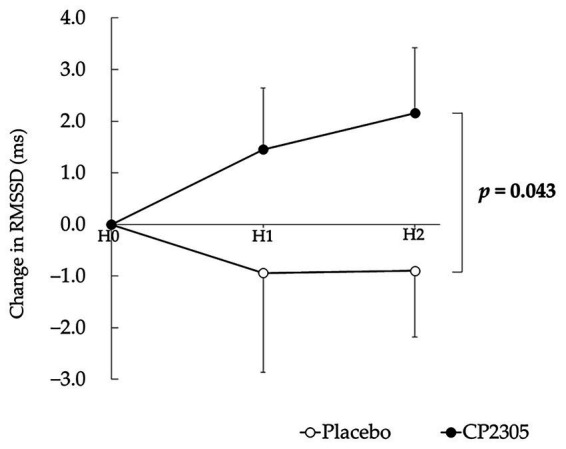
The effect of heat-treated CP2305 on HRV. Changes in RMSSD from baseline (H0) were compared between the placebo (*n* = 24) and heat-treated CP2305 (*n* = 24) intervention. Error bars represent standard errors. The *p*-value was calculated using a two-way ANOVA with repeated measures for the differences between the groups. HRV: Heart rate variability. RMSSD: Root mean square of successive differences.

The alpha power was assessed in the heat-treated CP2305 and placebo interventions (Supplementary Table 1). Changes in alpha power from the baseline (E0) are shown in Figure 4. The alpha power at Fz, Pz, O1, and O2 showed a significantly greater increase from baseline following the heat-treated CP2305 intervention than following the placebo intervention. No significant difference in alpha power changes was observed between the two conditions at the other four electrodes; however, alpha power at F3 following the heat-treated CP2305 intervention tended to increase compared with the placebo. Additionally, the effects on the beta power, which is known to be associated with tension and stress (38, 39), were evaluated. Changes in beta power from the baseline (E0) are shown in Table 2. The beta power changes at C4 under the heat-treated CP2305 intervention were significantly lower than those observed under the placebo intervention. No other differences were observed in beta power between the two conditions. Next, Next, RMSSD during the heat-treated CP2305 intervention was compared with that during the placebo intervention to assess its effect on parasympathetic activity (Supplementary Table 2). Figure 5 shows the changes in the RMSSD from the baseline (H0) under the two conditions. The change in RMSSD from baseline was significantly greater following heat-treated CP2305 intervention than following placebo intervention.

**Table 2:** The effects of heat-treated CP2305 on the beta oscillatory brain activity.

Electrodes	Interventions	E1 (dB) Mean ± SE	E2 (dB) Mean ± SE	E3 (dB) Mean ± SE	*p*-value
F3	Placebo	0.65 ± 0.29	0.56 ± 0.31	0.48 ± 0.36	0.360
	CP2305	0.63 ± 0.27	−0.10 ± 0.29	0.53 ± 0.41	
F4	Placebo	0.52 ± 0.25	0.16 ± 0.21	0.14 ± 0.29	0.284
	CP2305	0.23 ± 0.26	−0.30 ± 0.41	0.13 ± 0.55	
Fz	Placebo	0.20 ± 0.14	−0.05 ± 0.11	−0.13 ± 0.14	0.107
	CP2305	0.39 ± 0.16	0.00 ± 0.25	0.48 ± 0.47	
C3	Placebo	1.06 ± 0.51	0.90 ± 0.51	0.84 ± 0.55	0.161
	CP2305	0.83 ± 0.37	−0.05 ± 0.51	0.62 ± 0.64	
C4	Placebo	1.14 ± 0.49	1.20 ± 0.48	0.92 ± 0.41	0.013
	CP2305	0.58 ± 0.43	−0.36 ± 0.58	0.49 ± 0.60	
Pz	Placebo	0.68 ± 0.16	0.40 ± 0.15	0.39 ± 0.22	0.602
	CP2305	0.74 ± 0.23	0.18 ± 0.26	0.86 ± 0.50	
O1	Placebo	0.52 ± 0.29	−0.15 ± 0.41	−0.01 ± 0.30	0.534
	CP2305	0.63 ± 0.29	−0.16 ± 0.36	0.32 ± 0.41	
O2	Placebo	0.55 ± 0.29	0.21 ± 0.28	0.11 ± 0.35	0.472
	CP2305	0.77 ± 0.28	0.07 ± 0.29	0.60 ± 0.60	

Changes in beta power from baseline (E0) at eight electrodes were compared between the placebo (*n* = 22) and heat-treated CP2305 (*n* = 23) intervention. EEG data were obtained from the frontal (F3, F4, Fz), central (C3, C4), parietal (Pz), and occipital (O1, O2) areas. Data are presented as the mean ± standard error (SE). The *p*-values were calculated using two-way ANOVA with repeated measures for the differences between the groups.

### 3.3 Questionnaires

**Figure 6:**
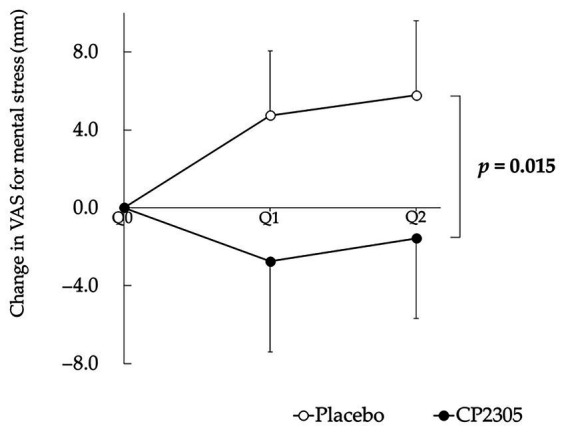
The effect of heat-treated CP2305 on the subjective stress. Changes in the VAS scores for mental stress from baseline (Q0) were compared between the placebo (*n* = 28) and heat-treated CP2305 (*n* = 28) intervention. Error bars represent standard errors. The *p*-value was calculated using a two-way ANOVA with repeated measures for the differences between the groups. VAS: visual analog scale.

The effects of heat-treated CP2305 on mental stress were evaluated using the VAS (Supplementary Table 3). Figure 6 shows the changes in the VAS scores for mental stress from the baseline (Q0) under the two conditions. Changes in mental stress scores during the heat-treated CP2305 intervention period were significantly lower than those during the placebo period. The results of the POMS2 assessment are presented in Table 3. Tension–anxiety (TA) scores decreased significantly more under the heat-treated CP2305 intervention than under the placebo. No other differences in mood status were observed between the two conditions.

**Table 3:** The effects of heat-treated CP2305 on the POMS2 scores.

Items	Interventions	Q1 Mean ± SE	Q2 Mean ± SE	*p*-value
AH	Placebo	−3.0 ± 0.8	−3.3 ± 0.9	0.745
	CP2305	−3.3 ± 0.9	−3.5 ± 1.0	
CB	Placebo	−0.4 ± 0.8	−1.3 ± 1.0	0.940
	CP2305	−0.9 ± 0.8	−0.7 ± 1.0	
DD	Placebo	−1.4 ± 0.5	−2.2 ± 0.8	0.714
	CP2305	−1.4 ± 0.8	−1.7 ± 0.9	
FI	Placebo	−1.6 ± 0.9	−3.3 ± 0.9	0.364
	CP2305	−2.0 ± 1.2	−1.3 ± 1.3	
TA	Placebo	−2.9 ± 0.9	−2.6 ± 1.0	0.022
	CP2305	−4.0 ± 0.9	−4.7 ± 0.9	
VA	Placebo	−2.8 ± 1.1	−3.5 ± 1.2	0.777
	CP2305	−2.9 ± 1.0	−3.9 ± 1.1	
F	Placebo	−2.2 ± 0.9	−3.4 ± 0.9	0.243
	CP2305	−1.0 ± 1.1	−2.8 ± 1.1	

Changes in the POMS2 scores from baseline (Q0) were compared between the placebo (*n* = 28) and heat-treated CP2305 (*n* = 28) interventions. Data are presented as the mean ± standard error (SE). The *p*-values were calculated using two-way ANOVA with repeated measures for the differences between the groups. AH, anger–hostility; CB, confusion–bewilderment; DD, depression–dejection; FI, fatigue–inertia; TA, tension–anxiety; VA, vigor–activity; F, friendliness.

### 3.4 *In vitro* assay

**Figure 7:**
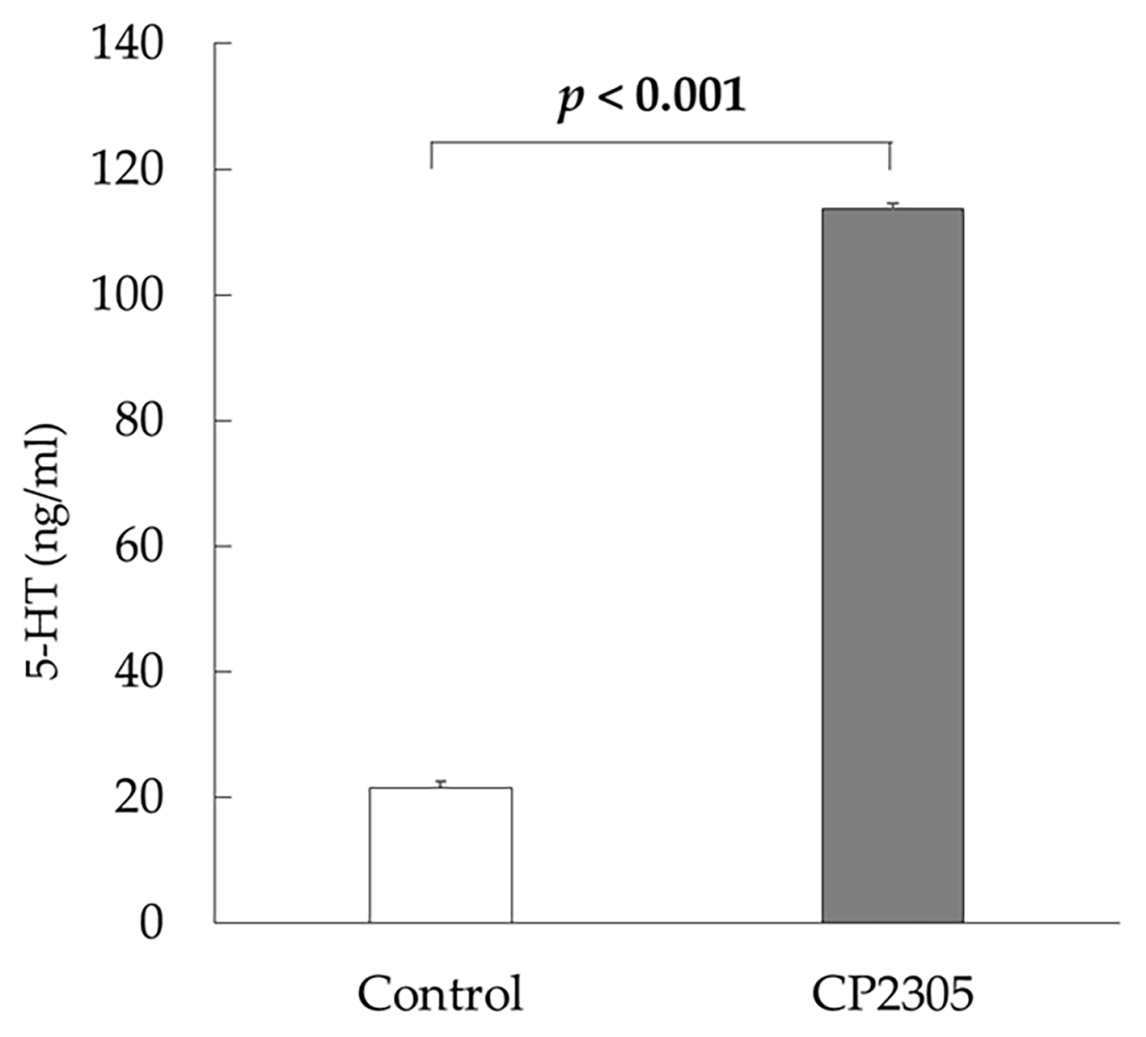
The effect of heat-treated CP2305 on the 5-HT secretion in RIN14B cell assay. The 5-HT concentration in the supernatant was measured. Error bars represent standard errors. The *p*-value was calculated using the Student’s *t*-test. 5-HT, 5-hydroxytryptamine.

To elucidate the potential effects of heat-treated CP2305 on the host intestinal epithelium, its effects were evaluated using an *in vitro* assay with RIN14B cells (35, 36). As shown in Figure 7, 5-HT levels in the culture supernatant were significantly higher under the heat-treated CP2305 condition compared with the control.

## 4 Discussion

This study explored the effects of heat-treated CP2305 intervention on brain oscillatory activity. Changes in alpha power 1 h after heat-treated CP2305 intervention were significantly greater in the frontal, parietal, and occipital regions than those observed with the placebo, suggesting that heat-treated CP2305 may support alpha oscillatory brain activity. In contrast, the effect of heat-treated CP2305 on beta power was observed only locally and was more limited than its effect on alpha power. To the best of our knowledge, no studies have shown that lactic acid bacteria affect alpha activity in the brain. Even probiotics have not been found to have such capacity, except for *Bifidobacterium longum* (*B. longum*) 1714™ (40), making CP2305 an interesting strain for brain activity modulation. While these two studies share the finding of post-stress alpha power enhancement, they differ in terms of bacterial viability, intervention duration, and stress modality. First, probiotic *B. longum* 1714™ may exert beneficial effects on mental health through temporary survival and metabolic activity in the gut, as well as through interactions with the gut microbiota. In contrast, since postbiotic heat-treated CP2305 is unable to survive in the gut or synthesize bioactive metabolites, it may exert its effects primarily through the host response to bacterial components. In addition, whereas the *B. longum* 1714™ study focused on emotional coping under social stress after long-term intake (40), our results highlight a rapid recovery following mental tasks after a single ingestion. Therefore, the present study may provide a complementary perspective on gut–brain stress modulation.

In this study, mental arithmetic and auditory oddball tasks were used as sets of mental tasks. The two mental tasks were intended to impose different dimensions of acute mental loads. The mental arithmetic task was designed to induce a high cognitive workload, whereas the auditory oddball task required sustained attention under monotonous conditions. The method of combining these two types of tasks as a mental load has been adopted in several randomized controlled trials (RCTs) to evaluate responses to acute stress (12, 41). Since at least 5 min of ECG data is recommended for accurate the HRV analysis (42), this study used 10 min of continuous ECG data recorded during the mental arithmetic task for HRV analysis. Changes in the RMSSD 1 h after heat-treated CP2305 intervention were significantly greater than those after the placebo intervention. RMSSD is widely used as an index of parasympathetic modulation and reflects the integrity of vagally mediated autonomic control (30). Lower RMSSD values have been consistently reported in patients with depressive and anxiety disorders, indicating reduced parasympathetic activity (43–45). In contrast, higher RMSSD values observed in healthy individuals are generally considered to reflect greater beat-to-beat HRV and a more adaptable autonomic nervous system (ANS), which is associated with better stress regulation and overall health (46, 47). In this context, the changes in RMSSD observed after heat-treated CP2305 intervention suggest the maintenance of parasympathetic activity during mental tasks.

The present study demonstrated that a single dose of heat-treated CP2305 elicited measurable changes in brain oscillatory activity within 60 min; traditional absorption theories cannot fully explain the effects that appear within 60 min. This relatively rapid EEG response is thought to be more consistent with sensory-mediated gut–brain communication, in which gastrointestinal stimulation engages vagal afferent signaling that rapidly conveys information to central autonomic and brain networks (48). Notably, changes in parasympathetic activity occur within the same short time window. In the context of the GBA, neural signaling via vagal afferent pathways represents one of the most plausible routes capable of transmitting intestinal signals to the brain on such a short timescale. The single-dose design, rapid onset of effects, and concomitant increase in RMSSD suggest, but do not prove, that vagal-mediated gut–brain signaling may contribute to the observed effects of heat-treated CP2305.

Signals conveyed to the nucleus tractus solitarius (NTS) via afferent vagal pathways are further transmitted to higher brain regions through multiple ascending circuits (49). In particular, the NTS output is relayed to the cerebral cortex via thalamocortical projections, while parallel projections to the hypothalamus contribute to the modulation of ANS activity (49, 50). Although the precise mechanisms underlying alpha rhythm generation remain incompletely understood, converging evidence indicates that the thalamus plays a central role in shaping cortical alpha oscillations (5, 6). Activation of thalamocortical loops has been shown to enhance alpha-band power, particularly in the occipital cortex (7). In this context, gut-derived sensory signaling reaching the NTS via vagal afferents may indirectly influence cortical alpha activity through thalamic pathways, while simultaneously modulating parasympathetic outflow reflected in HRV measures. Taken together, these observations are consistent with a brainstem–thalamic pathway that may contribute to the association between gastrointestinal stimulation and changes in autonomic activity and cortical alpha oscillations.

To gain insight into the potential effects of heat-treated CP2305 on the host intestinal epithelium, we evaluated its impact using RIN14B cells, a well-established rat enterochromaffin cell (ECC) model. This *in vitro* analysis demonstrated that heat-treated CP2305 administration significantly increased 5-HT levels in culture supernatants. Nevertheless, caution is warranted when extrapolating 5-HT secretion observed in an *in vitro* ECC model to human neurophysiological outcomes, such as cortical alpha activity or heart rate variability, given the multiple biological gaps that exist across species, experimental conditions, and sites of action. 5-HT is a key neurotransmitter, approximately 90–95% of which is produced and stored within ECCs (51). 5-HT secreted from ECCs is known to stimulate the terminals of vagal afferent neurons (52), and this sensory information is subsequently relayed as electrical signals to the NTS (53). The NTS serves as a primary integration center for visceral sensory inputs and plays a critical role in downstream autonomic and central nervous system regulation (49, 50). Taken together, although the present *in vitro* findings do not provide direct evidence linking ECC-derived 5-HT release to changes in cortical or autonomic activity in humans, these observations are consistent with the possibility that brainstem–thalamic pathways contribute to the association between gastrointestinal stimulation and changes in autonomic activity and cortical alpha oscillations.

Finally, changes in subjective psychological features were also evaluated using questionnaires since physiological tension and relaxation can affect mood states such as anxiety, confusion, or vigor (54, 55). In this study, changes in mental stress levels following test tablets intake were evaluated using the VAS. The VAS score alterations significantly differed between the two conditions. While the VAS scores with the placebo increased after the mental tasks, those with heat-treated CP2305 showed almost no change within 60 min. This suggests that heat-treated CP2305 may alleviate the subjective stress caused by mental tasks. In a previous study, we found that 24-week heat-treated CP2305 administration decreased stressful irritability, as measured by the VAS in healthy young adults exposed to chronic stress (21). The results obtained under temporary stress conditions in the present study are consistent with the responses to chronic stress observed in our previous study.

POMS2 was used as an appropriate questionnaire to evaluate temporary mood changes (32). It consists of five negative and two positive mood states and can be used to screen for mental disorders, evaluate the effects of food ingredients on mood changes, and verify the efficacy of relaxation techniques. In this study, no significant differences in mood changes assessed by the POMS2, except for tension–anxiety (TA), were observed between the two conditions. The lack of significant changes in most POMS2 domains suggests a limited impact on mood status beyond tension-anxiety. However, the TA scores under the heat-treated CP2305 condition were significantly lower than those under the placebo condition. Higher TA scores indicate greater tension and anxiety. Therefore, these results suggest that heat-treated CP2305 may improve tension and anxiety. A decrease in the TA scores has also been observed after forest bathing or aromatherapy, which can activate the parasympathetic nervous system (54, 56, 57). Furthermore, both the TA scores and subjective stress levels scaled by the VAS decreased after L-theanine administration, which can increase alpha power in the brain (26, 41, 58). Tension activates the sympathetic nervous system and increases blood pressure, heart rate, and respiratory rate. Conversely, activation of the parasympathetic nervous system decreases blood pressure, heart rate, and respiratory rate. In conclusion, improvements in TA scores may be related to the activation of the parasympathetic nervous system.

The concept of psychobiotics was originally proposed to describe live microorganisms that confer mental health benefits through interactions along the microbiota–gut–brain axis, including neural, endocrine, and immune pathways (59). However, more recent discussions have broadened this concept to include non-viable microorganisms and bacterial preparations that exert psychotropic effects, independent of microbial viability (60). In addition to the acute neurophysiological effects observed in the present study, previous clinical trials have shown that heat-treated CP2305 improves sleep quality and alleviates stress-related symptoms (18, 19, 21, 22). Collectively, these findings suggest that heat-treated CP2305 exerts beneficial effects across multiple domains relevant to mental health. From this expanded perspective, heat-treated CP2305 can be conceptually placed within the psychobiotics framework. Importantly, psychobiotics differ from conventional neuroactive drugs in several clinically relevant aspects. Rather than targeting specific receptors or neurotransmitter systems directly, psychobiotics modulate host physiology via endogenous regulatory pathways such as the ANS and gut–brain signaling, which may reduce the risk of adverse effects, dependence, or tolerance (61). In this context, non-viable psychobiotics, such as heat-treated CP2305, represent a promising complementary approach for supporting mental health, combining neurophysiological relevance with favorable safety and tolerability profiles.

This study had some limitations that should be considered. First, EEG has limitations in measuring neural activity in deep brain regions because of its poor spatial resolution and the attenuation of signals from deeper structures. Thus, it is not possible to pinpoint the precise location of signals originating from deeper areas, such as the hippocampus or amygdala. Further studies using neuroimaging techniques, such as Near-Infrared Spectroscopy (NIRS) or functional Magnetic Resonance Imaging (fMRI), may eliminate this limitation to understand the impact of heat-treated CP2305 on brain function. Second, since EEG data were collected using eight electrodes, it took over 30 min to attach them to the scalp. Contrary to expectations, the TA scores on the POMS2 under the placebo condition did not increase after the mental tasks. The participants may have already been in a state of heightened tension or anxiety before intervention because of the lengthy procedure required to attach the electrodes. Third, the study population was restricted to healthy adults aged 20–44 years. While this relatively homogeneous population helps to reduce inter-individual variability, extrapolation of the present findings to older individuals, adolescents, or clinical populations should be made cautiously, as stress reactivity may differ across age groups and health conditions. Therefore, future studies that include broader age ranges and relevant clinical populations will be needed to confirm the generalizability of these findings. Fourth, the overall sample size was modest. While the planned enrollment was based on an *a priori* sample size estimation derived from previous work and was sufficient to detect effects of the expected magnitude, the study may have limited statistical power to detect subtle effects, and smaller changes may have gone undetected. Fifth, some participants were excluded from the EEG and HRV analyses due to data quality considerations (e.g., missing trigger logs related to technical issues and abnormal signal patterns). Although these exclusions were unavoidable given the data quality constraints, they could introduce selection bias and reduce the effective sample size for certain outcomes. In addition to increasing sample size, future studies may help minimize avoidable data loss and potential bias by further standardizing data acquisition procedures and refining pre-assessment and screening procedures. The elucidation of the mechanisms by which heat-treated CP2305 modulates brain oscillatory activity is an important next step. Additionally, a recent study showed that alpha power is linked to working memory (62). Ingredients that increase alpha power may have attractive benefits, such as improved working memory, and are therefore promising for future applications.

In conclusion, this study provides evidence that a single dose of heat-treated CP2305 consumption is associated with brain oscillatory activity, autonomic nerve regulation, and subjective stress responses. Although further research is required to elucidate the underlying mechanisms, the concurrent modulation of cortical alpha activity, parasympathetic indices, and stress-related subjective measures suggests the relevance of gut-brain communication in these effects. These findings suggest that heat-treated CP2305 could be a promising functional food ingredient that supports mental health, while highlighting the importance of extension in future studies.

## Data Availability

The original contributions presented in the study are included in the article/Supplementary material, further inquiries can be directed to the corresponding author.
