# Supplementary material for: Effects of heat-treated Lactobacillus gasseri CP2305 on brain oscillatory activity and mental stress in healthy adults: a double-blind, randomized, crossover, placebo-controlled trial
Source: Front Nutr. 2026 May 28;13:1796729. doi: 10.3389/fnut.2026.1796729 (PMC13253227; doi:10.3389/fnut.2026.1796729)
Supplement: Supplementary file 1 [file Table_1.docx]

# Supplementary Table 1. Alpha power values (dB) at each time point for the placebo and heat-treated CP2305 interventions.

| **Electrodes** | **Placebo (Mean ± SE)** | | | | **CP2305 (Mean ± SE)** | | | |
| --- | --- | --- | --- | --- | --- | --- | --- | --- |
|  | **E0** | **E1** | **E2** | **E3** | **E0** | **E1** | **E2** | **E3** |
| F3 | 12.0 ± 0.8 | 12.5 ± 0.8 | 12.0 ± 0.8 | 12.4 ± 0.9 | 11.6 ± 0.8 | 13.0 ± 0.7 | 12.3 ± 0.8 | 12.9 ± 0.8 |
| F4 | 12.0 ± 0.8 | 12.6 ± 0.8 | 12.0 ± 0.9 | 12.4 ± 0.9 | 11.7 ± 0.8 | 13.0 ± 0.7 | 12.4 ± 0.8 | 12.9 ± 0.8 |
| Fz | 12.2 ± 0.8 | 12.8 ± 0.9 | 12.2 ± 0.9 | 12.5 ± 0.9 | 11.7 ± 0.8 | 13.1 ± 0.8 | 12.5 ± 0.8 | 13.0 ± 0.8 |
| C3 | 9.6 ± 0.7 | 10.0 ± 0.7 | 9.6 ± 0.8 | 10.0 ± 0.8 | 9.2 ± 0.7 | 10.4 ± 0.6 | 9.7 ± 0.7 | 10.4 ± 0.7 |
| C4 | 9.7 ± 0.7 | 10.3 ± 0.8 | 9.9 ± 0.8 | 10.2 ± 0.8 | 9.7 ± 0.8 | 10.8 ± 0.7 | 10.2 ± 0.8 | 10.8 ± 0.8 |
| Pz | 12.9 ± 0.8 | 13.1 ± 1.0 | 12.7 ± 0.9 | 12.9 ± 0.9 | 12.1 ± 0.9 | 13.2 ± 0.8 | 12.8 ± 0.8 | 13.4 ± 0.8 |
| O1 | 16.7 ± 0.9 | 17.3 ± 1.0 | 16.5 ± 1.1 | 17.0 ± 1.0 | 16.4 ± 1.0 | 18.0 ± 0.9 | 17.2 ± 0.9 | 17.5 ± 0.9 |
| O2 | 16.6 ± 0.8 | 17.2 ± 0.9 | 16.6 ± 0.9 | 16.9 ± 1.0 | 16.1 ± 0.9 | 17.6 ± 0.9 | 16.9 ± 0.9 | 17.4 ± 0.9 |

EEG data were obtained from the frontal (F3, F4, Fz), central (C3, C4), parietal (Pz), and occipital (O1, O2) areas. Data are presented as the mean ± standard error (SE).

# Supplementary Table 2. RMSSD values (ms) at each time point for the placebo and heat-treated CP2305 interventions.

| **Items** | **Placebo (Mean ± SE)** | | | **CP2305 (Mean ± SE)** | | |
| --- | --- | --- | --- | --- | --- | --- |
|  | **H0** | **H1** | **H2** | **H0** | **H1** | **H2** |
| RMSSD | 29.7 ± 3.3 | 31.2 ± 4.7 | 28.4 ± 2.5 | 33.6 ± 4.5 | 35.0 ± 4.5 | 33.1 ± 3.7 |

Data are presented as the mean ± standard error (SE). RMSSD: root mean square of successive differences.

# Supplementary Table 3. VAS scores (mm) for mental stress at each time point for the placebo and heat-treated CP2305 interventions.

| **Items** | **Placebo (Mean ± SE)** | | | **CP2305 (Mean ± SE)** | | |
| --- | --- | --- | --- | --- | --- | --- |
|  | **Q0** | **Q1** | **Q2** | **Q0** | **Q1** | **Q3** |
| Mental stress | 29.5 ± 4.5 | 34.2 ± 4.5 | 35.3 ± 4.6 | 38.6 ± 4.4 | 35.8 ± 4.4 | 37.0 ± 4.1 |

The VAS was used with 0 mm anchored to “low mental stress” and 100 mm to “high mental stress”. The VAS scores were calculated based on the distance from the 0 mm position. Data are presented as the mean ± standard error (SE). VAS: visual analogue scale.
